# Supplementary material for: The high incidence of severe adverse events due to pyrazinamide in elderly patients with tuberculosis
Source: PLoS One. 2020 Jul 21;15(7):e0236109. doi: 10.1371/journal.pone.0236109 (PMC7373258; doi:10.1371/journal.pone.0236109)
Supplement: S1 Table — (DOCX) [file pone.0236109.s001.docx]

**Table S1.** Serious adverse events of first-line anti-TB medication in patients who died or discontinued treatment.

|  | Total (n = 169) | Death (n = 67) | Early discontinuation (n = 102) |
| --- | --- | --- | --- |
| Any types of SAEs | 75 (44.4%) | 19 (28.4%) | 56 (54.9%) |
| Causative drugs |  |  |  |
| PZA | 28 (37.3%) | 7 (36.8%) | 21 (20.6%) |
| EMB | 13 (17.3%) | 2 (10.5%) | 11 (10.8%) |
| RIF | 9 (12.0%) | 1 (5.3%) | 8 (7.8%) |
| INH | 3 (4.0%) | 1 (5.3%) | 2 (2.0%) |
| Unknown | 25 (33.3%) | 8 (42.1%) | 17 (30.4%) |

TB, tuberculosis; SAE, severe adverse event; PZA, pyrazinamide; EMB, ethambutol; RIF, rifampin; INH, isoniazid

Data are reported as mean ± standard deviation and numbers (%).
