# Supplementary material for: The high incidence of severe adverse events due to pyrazinamide in elderly patients with tuberculosis
Source: PLoS One. 2020 Jul 21;15(7):e0236109. doi: 10.1371/journal.pone.0236109 (PMC7373258; doi:10.1371/journal.pone.0236109)
Supplement: S2 Table — (DOCX) [file pone.0236109.s002.docx]

**Table S2.** Baseline characteristics of patients with pyrazinamide-associated hepatotoxicity

| Variables | Hepatotoxicity | | |
| --- | --- | --- | --- |
|  | +, N=101 | ‒, N=126 | *P* value |
| Age (year) | 53.8±19.6 | 56.9±17.6 | 0.210 |
| Sex, male (%) | 55 (54.1) | 61 (48.4) | 0.365 |
| Tuberculosis |  |  | 0.245 |
| Pulmonary | 91 (90.1) | 107 (84.9) |  |
| Extrapulmonary | 10 (9.9) | 19 (15.1) |  |
| Initial diagnosis |  |  | 0.308 |
| Sputum AFB | 34 (35.4) | 35 (28.9) |  |
| TB-PCR | 62 (64.6) | 86 (71.1) |  |
| Comorbidities |  |  |  |
| DM | 9 (8.9) | 21 (16.7) | 0.086 |
| Renal insufficiency | 1 (1.0) | 3 (2.4) | 0.631 |
| Long-term steroid | 0 (0.0) | 0 (0.0) | - |
| Smoking^a^ |  |  | 0.559 |
| Never | 54 (62.1) | 62 (57.4) |  |
| Ex- or current | 33 (37.9) | 46 (42.6) |  |
| Alcohol^b^ |  |  | 0.983 |
| Never/social | 25 (92.6) | 51 (92.7) |  |
| Heavy | 2 (7.4) | 4 (7.3) |  |
| HBs Ag (+) | 4 (5.6) | 2 (2.6) | 0.428 |
| Anti HCV (+) | 0 (0.0) | 0 (0.0) | - |
| Liver function test |  |  |  |
| AST | 25.8±21.8 | 22.8±10.8 | 0.278 |
| ALT | 19.9±13.1 | 19.3±13.9 | 0.766 |
| Treatment duration (mo) | 9.7±3.2 | 8.9±3.5 | 0.063 |

AFB, Acid-fast blue; TB, tuberculosis; PCR, polymerase chain reaction; DM, diabetes mellitus; mo, months

^a^Data were not recorded for 32 (14.1%) patients

^b^Data were not recorded for 145 (63.9%) patients

Data are reported as mean ± standard deviation and numbers (%).
