# Supplementary material for: The high incidence of severe adverse events due to pyrazinamide in elderly patients with tuberculosis
Source: PLoS One. 2020 Jul 21;15(7):e0236109. doi: 10.1371/journal.pone.0236109 (PMC7373258; doi:10.1371/journal.pone.0236109)
Supplement: S3 Table — (DOCX) [file pone.0236109.s003.docx]

**Table S3.** Baseline characteristics of patients with pyrazinamide-associated GI intolerance

| Variables | GI intolerance | | |
| --- | --- | --- | --- |
|  | +, N=54 | ‒, N=173 | *P* value |
| Age (year) | 61.8±18.8 | 53.5±18.0 | 0.004 |
| Sex, male (%) | 20 (37.0) | 96 (55.5) | 0.018 |
| Tuberculosis |  |  | 0.148 |
| Pulmonary | 44 (81.5) | 154 (89.0) |  |
| Extrapulmonary | 10 (18.5) | 19 (11.0) |  |
| Initial diagnosis |  |  | 0.874 |
| Sputum AFB | 17 (32.7) | 52 (31.5) |  |
| TB-PCR | 35 (67.3) | 113 (68.5) |  |
| Comorbidities |  |  |  |
| DM | 15 (27.8) | 15 (8.7) | <0.001 |
| Renal insufficiency | 0 (0.0) | 4 (2.3) | 0.575 |
| Long-term steroid | 0 (0.0) | 0 (0.0) | - |
| Smoking^a^ |  |  | 0.159 |
| Never | 30 (69.8) | 86 (56.6) |  |
| Ex- or current | 13 (30.2) | 66 (43.4) |  |
| Alcohol^b^ |  |  | 0.266 |
| Never/social | 29 (96.7) | 47 (90.4) |  |
| Heavy | 1 (3.3) | 5 (9.6) |  |
| HBs Ag (+) | 0 (0.0) | 6 (5.1) | 0.342 |
| Anti HCV (+) | 0 (0.0) | 0 (0.0) | - |
| Liver function test |  |  |  |
| AST | 21.8±7.6 | 24.8±18.5 | 0.375 |
| ALT | 17.5±10.1 | 20.2±14.2 | 0.325 |
| Treatment duration (mo) | 8.6±2.7 | 9.4±3.5 | 0.068 |

GI, gastrointestine; AFB, Acid-fast blue; TB, tuberculosis; PCR, polymerase chain reaction; DM, diabetes mellitus;

^a^Data were not recorded for 32 (14.1%) patients

^b^Data were not recorded for 145 (63.9%) patients

Data are reported as mean ± standard deviation and numbers (%).
