# Supplementary material for: The high incidence of severe adverse events due to pyrazinamide in elderly patients with tuberculosis
Source: PLoS One. 2020 Jul 21;15(7):e0236109. doi: 10.1371/journal.pone.0236109 (PMC7373258; doi:10.1371/journal.pone.0236109)
Supplement: S4 Table — (DOCX) [file pone.0236109.s004.docx]

**Table S4.** Baseline characteristics of patients with pyrazinamide-associated skin reaction

| Variables | Skin rash | | |
| --- | --- | --- | --- |
|  | +, N=36 | ‒, N=191 | *P* value |
| Age (year) | 51.6±14.9 | 56.2±19.1 | 0.109 |
| Sex, male (%) | 20 (55.6) | 96 (50.3) | 0.560 |
| Tuberculosis |  |  | 0.446 |
| Pulmonary | 30 (83.3) | 168 (88.0) |  |
| Extrapulmonary | 6 (16.7) | 23 (12.0) |  |
| Initial diagnosis |  |  | 0.468 |
| Sputum AFB | 9 (26.5) | 60 (32.8) |  |
| TB-PCR | 25 (73.5) | 123 (67.2) |  |
| Comorbidities |  |  |  |
| DM | 2 (5.6) | 28 (14.7) | 0.183 |
| Renal insufficiency | 1 (2.8) | 3 (1.6) | 0.501 |
| Long-term steroid | 0 (0.0) | 0 (0.0) | - |
| Smoking^a^ |  |  | 0.250 |
| Never | 17 (50.0) | 99 (61.5) |  |
| Ex- or current | 17 (50.0) | 62 (38.5) |  |
| Alcohol^b^ |  |  | 0.978 |
| Never/social | 13 (92.9) | 63 (92.6) |  |
| Heavy | 1 (7.1) | 5 (7.4) |  |
| HBs Ag (+) | 1 (4.2) | 5 (4.0) | 1.000 |
| Anti HCV (+) | 0 (0.0) | 0 (0.0) | - |
| Liver function test |  |  |  |
| AST | 22.8±14.7 | 24.4±17.3 | 0.659 |
| ALT | 18.3±13.3 | 19.8±13.5 | 0.608 |
| Treatment duration (mo) | 9.2±4.4 | 9.3±3.1 | 0.893 |

AFB, Acid-fast blue; TB, tuberculosis; PCR, polymerase chain reaction; DM, diabetes mellitus;

^a^Data were not recorded for 32 (14.1%) patients

^b^Data were not recorded for 145 (63.9%) patients

Data are reported as mean ± standard deviation and numbers (%).
