# Supplementary material for: The high incidence of severe adverse events due to pyrazinamide in elderly patients with tuberculosis
Source: PLoS One. 2020 Jul 21;15(7):e0236109. doi: 10.1371/journal.pone.0236109 (PMC7373258; doi:10.1371/journal.pone.0236109)
Supplement: S5 Table — (DOCX) [file pone.0236109.s005.docx]

**Table S5.** Baseline characteristics of patients with pyrazinamide-associated arthropathy

| Variables | Arthropathy | | |
| --- | --- | --- | --- |
|  | +, N=27 | ‒, N=200 | *P* value |
| Age (year) | 53.1±16.2 | 55.8±18.8 | 0.475 |
| Sex, male (%) | 16 (59.3) | 100 (50.0) | 0.416 |
| Tuberculosis |  |  | 0.216 |
| Pulmonary | 26 (96.3) | 172 (86.0) |  |
| Extrapulmonary | 1 (3.7) | 28 (14.0) |  |
| Initial diagnosis |  |  | 0.658 |
| Sputum AFB | 7 (26.9) | 62 (32.5) |  |
| TB-PCR | 19 (73.1) | 129 (67.5) |  |
| Comorbidities |  |  |  |
| DM | 2 (7.4) | 28 (14.0) | 0.343 |
| Renal insufficiency | 1 (3.7) | 3 (1.5) | 0.466 |
| Long-term steroid | 0 (0.0) | 0 (0.0) | - |
| Smoking^a^ |  |  | 0.501 |
| Never | 12 (52.2) | 104 (60.5) |  |
| Ex- or current | 11 (47.8) | 68 (39.5) |  |
| Alcohol^b^ |  |  | 0.094 |
| Never/social | 6(75.0) | 70 (94.6) |  |
| Heavy | 2 (25.0) | 4 (5.4) |  |
| HBs Ag (+) | 1 (5.9) | 5 (3.6) | 0.664 |
| Anti HCV (+) | 0 (0.0) | 0 (0.0) | - |
| Liver function test |  |  |  |
| AST | 25.1±10.7 | 24.0±17.5 | 0.806 |
| ALT | 26.6±20.2 | 18.7±12.2 | 0.131 |
| Treatment duration (mo) | 8.9±3.6 | 9.3±3.3 | 0.614 |

AFB, Acid-fast blue; TB, tuberculosis; PCR, polymerase chain reaction; DM, diabetes mellitus;

^a^Data were not recorded for 32 (14.1%) patients

^b^Data were not recorded for 145 (63.9%) patients

Data are reported as mean ± standard deviation and numbers (%).
