# Supplementary material for: The high incidence of severe adverse events due to pyrazinamide in elderly patients with tuberculosis
Source: PLoS One. 2020 Jul 21;15(7):e0236109. doi: 10.1371/journal.pone.0236109 (PMC7373258; doi:10.1371/journal.pone.0236109)
Supplement: Attachment — Submitted filename: Response_to_comments_R1.docx [file pone.0236109.s006.docx]

Seyed Ehtesham Hasnain, PhD

Editor

*PLOS ONE*

Dear Prof. Hasnain:

We would like to thank you and the reviewers for your comments regarding our manuscript, titled “**The high incidence of severe adverse reactions to pyrazinamide in elderly patients with tuberculosis**” (manuscript ID: PONE-D-20-09108), which we now wish to resubmit for consideration for publication. We believe the new version of our manuscript has been significantly improved after the incorporation of your valuable suggestions.

The manuscript has been revised in accordance with your suggestions, and our point-by-point response to the reviewers’ comments is provided below. Please note that we have revised our title to reduce the chances of misinterpretation as indicated by the reviewer.

Original manuscript title:

“The high incidence of severe adverse reactions to pyrazinamide in elderly patients with tuberculosis”

Revised manuscript title:

“The high incidence of severe adverse events due to pyrazinamide in elderly patients with tuberculosis”

We hope that, with these changes, the manuscript is now deemed suitable for publication in *PLOS ONE* as a *Research Article*.

Sincerely,

Jae Ho Lee, MD, PhD

Division of Pulmonary and Critical Care Medicine, Department of Internal Medicine, Seoul National University Bundang Hospital

82 Gumi-ro 173 Beon-gil, Bundang-gu, Seongnam-si, Gyeonggi-do, 13620, Korea

Tel.: 031-787-7011

Fax: 031-787-2204

E-mail: jhlee7@snubh.org

**Responses to Reviewer 1:**

1. The methodology of how causality to pyrazinamide was assessed must be described in detail.

🡪 Thank you for your comment. We have added the following sentences in the method section of the revised manuscript.

“The causative drug was determined based on the disappearance of the SAEs upon the withdrawal of the most suspected drug without re-challenge or the recurrence of the same SAE with drug re-challenge. In cases of withdrawal, the decision regarding the discontinuation of the suspected drug was made by the attending physicians.”

(Page 5–6, line 107–111 in the revised manuscript)

2. Number of serious adverse drug reactions identified by drug withdrawal (dechallenge) and the by drug re-introduction (rechallenge) must be presented and discussed.

🡪 Thank you for your insightful comment. We completely agree with your opinion.

Among the 227 patients who experienced SAEs due to PZA, the numbers of cases identified by de-challenge and re-challenge were 188 (82.8%) and 39 (17.2%), respectively. According to the types of SAEs, physicians were more likely to withdraw PZA in cases of hepatotoxicity and GI intolerance. The results are shown in the following table.

|  | Total | De-challenge | Re-challenge |
| --- | --- | --- | --- |
| Total | 227 | 188 (82.8%) | 39 (17.2%) |
| Hepatotoxicity | 101 | 85 (84.2%) | 16 (15.8%) |
| GI intolerance | 54 | 45 (83.3%) | 9 (16.7%) |
| Cutaneous adverse reactions | 36 | 23 (63.9%) | 13 (36.1%) |
| Arthropathy | 27 | 27 (100%) | 0 (0.0%) |
| Others | 9 | 8 (88.9%) | 1 (11.1%) |

We have added the following sentence in the result section.

“Among the 227 patients who experienced SAEs due to PZA, 188 (82.8%) and 39 (17.2%) cases were identified by withdrawal and re-challenge, respectively.”

(Page 7, line 139–141 in the revised manuscript)

Additionally, we have added the following sentence in the discussion section in the revised manuscript.

“Third, the high confirmation rate as a causative drug by only de-challenging might suggest that physicians preferentially discontinued PZA because it was not a maintenance drug and as previously reported side effects were high.”

(Page 13, line 205–208 in the revised manuscript)

In the original manuscript, we had stated that 26 patients suffered from arthropathy. However, one patient was inadvertently missed. Thus, we have corrected the number in the following sentence as well as in **Table 2** and **Table S5** in the revised manuscript. We apologize for the error.

“Arthropathy, a PZA-specific SAE, occurred in 27 patients.”

(Page 10, line 163–164 in the revised manuscript)

3. The acronym SARS and SARs have been used for serious adverse drug reactions. In view of the world-wide pandemic related to the SARS-Cov-2 virus infection, the acronym used by the authors has every chance to be misinterpreted. It is suggested that the authors modify this acronym

🡪 Thank you for your comment.

We have changed the term “severe adverse reactions (SARs)” to “severe adverse events (SAEs)” throughout the revised manuscript.

4. The 'Introduction' is not well developed. Why the present study was necessary to be conducted with the available published literature is not explained.

🡪 Thank you for your comment. We have rewritten the introduction and have added references as follows.

“Considering that older age is a notable risk factor for SAEs (9, 16, 17) owing to several comorbidities (18) or polypharmacy, healthcare providers who manage TB must exercise caution while selecting suitable anti-TB drugs and doses and monitor compliance. According to previous studies, pyrazinamide causes the most SAEs among first-line anti-TB drugs (9, 10, 19). However, there is limited information regarding the safety of PZA in elderly patients (20, 21). In particular, the incidence and types of SAEs and their relationship with patient age have not been thoroughly investigated previously. Therefore, we aimed to demonstrate the relationship of age and type of SAE to PZA.”

(Page 4, line 72–79 in the revised manuscript)

5. The Discussion section contains repetition of the results. The discussion needs to be more pointed towards the issues identified from the results of the study. A loosely written discussion reads like a review of literature.

🡪 Thank you for your comment. We fully agree with your opinion.

We have deleted the sentence that repeats the results in the discussion section.

“Among the SARs to PZA, hepatotoxicity was the most common adverse event, followed by GI intolerance, cutaneous adverse reactions, and arthropathy.”

Additionally, we have revised the following sentence.

“In this study, adverse effects that led to the interruption or discontinuation of medication occurred in 16.4% of patients, and PZA was the most common causative drug.”

(Page 13, line 193–194 in the revised manuscript)

“Although the exact mechanism of hepatotoxicity is not fully understood, drug metabolism rather than the peak drug level might contribute to the development of hepatotoxicity to PZA. For instance, an animal study showed that two PZA metabolites, pyrazinoic acid and 5-hydroxypyrazinoic acid, are responsible for hepatotoxicity (25). In contrast, several studies measuring the serum PZA level revealed that the occurrence of hepatotoxicity was not significantly related to the drug concentration (19, 26).”

(Page 13–14, line 215–220 in the revised manuscript)

“Overall, the incidence of cutaneous adverse reactions due to anti-TB medication varies across studies, and the results for the most common causative drugs also differ (9, 32, 33). For instance, Marra et al. reported that an EMB-containing regimen tends to increase the risk of cutaneous adverse reactions, though there is no statistically significant difference (32). Similarly, in a study by Kim et al., 16.8% of patients experienced severe cutaneous adverse reactions, and SAEs due to EMB were more common (6.2%) than those to PZA (3.7%) (33). In contrast, Yee et al. reported that the incidence of severe rash is mostly associated with PZA (9).”

(Page 14, line 243–249 in the revised manuscript)

6. Please highlight the new knowledge presented in the paper.

🡪 Thank you for your comment. We have revised the conclusion section of the revised manuscript as follows.

“The incidence of SAEs due to PZA was 9.2%, and old age was a notable risk factor for SAE occurrence. In the majority of cases, a causal relationship was identified by the withdrawal of PZA, even without re-challenge. Therefore, the importance of the early recognition of SAEs and decisions regarding whether to discontinue should be emphasized to ensure effective treatment compliance, especially in elderly patients.”

(Page 16, line 271–275 in the revised manuscript)

**Responses to Reviewer 2:**

The study by Kwon et al. is conducted very well in retrospectively especially on both pulmonary and extra-pulmonary TB patients; data is well organized and presented. The manuscript is well written and understandable with SARs occurred with PZA, most commonly hepatotoxicity and GI intolerance in elderly patients. Following are some suggestions which can make the manuscript stronger and beneficial for the readers:

**Abstract:**
1. Abbreviation of TB was maintained for “first-line anti-tuberculosis”, correct it.

🡪 We have corrected the abbreviation of TB in the revised manuscript.

(Page 3, line 40 in the revised manuscript)

2. Specify the name of first-line anti-tuberculosis drugs.

🡪 We have added the names of the first-line anti-tuberculosis drugs in the abstract.

“The medical records of patients with tuberculosis (TB) treated with PZA-containing regimens including first-line drugs—ethambutol, rifampicin, and isoniazid—from January 2003 to June 2016 were reviewed.”

(Page 3, line 40–42 in the revised manuscript)

3. Conclusive statement should be modified to give good interpretation to the reader.

🡪 Thank you for your insightful comment. We have revised the conclusion of the abstract as follows.

“PZA was the most common drug associated with SAEs among the first-line anti-TB drugs, and old age was an independent factor for SAE occurrence. This study suggests that the early recognition of whether the causative agent is PZA may improve effective treatment compliance, particularly in elderly patients.”

(Page 3, line 52–55 in the revised manuscript)

**Method:** It observed that author/s ware collected the data very precisely. However, required some information to improve manuscript like:

4. Author should specify the follow-up criteria for EPTB patients during SARs.

🡪 Thank you for your comment. Unfortunately, the follow-up of patients with TB experiencing SAEs is not performed according to set protocols in our center. Additionally, the follow-up schedule varies according to the attending physicians. Nonetheless, a few pulmonology experts treated and managed the SAEs consistently.

We analyzed the first follow-up date from the time of the occurrence of the SAEs. The median time from the recognition of the SAE to the first follow-up was 9.0 days (interquartile range, 7.0–14.0 days). There was no statistically significant difference between patients with pulmonary and extrapulmonary TB.

We have added the following sentences in the result and discussion sections, instead of the method section.

“The median duration of time between the recognition of the SAEs and the first follow-up was 9.0 days (interquartile range, 7.0–14.0 days).”

(Page 7, line 133–134 in the revised manuscript)

“Additionally, there were no set protocols for following or managing SAEs.”

(Page 15, line 255–256 in the revised manuscript)

**Results:**
5. In patient history, alcoholism and smoker status of patient as well as house hold contact should be noted if possible. Because these habits are strongly associated with TB disease and attributed to high risk of SARs in elderly patients.

🡪 Thank you for your comment. First, information on household contacts is not routinely collected in our institution. Additionally, owing to the retrospective nature of the study, we cannot provide these details. We apologize for this lack of data. In addition, as we mentioned, there was a large amount of data on alcohol consumption that was missing (page 18, line 256 in the revised manuscript). Although these data were missing, we have revised Table 1 and Table S2-S5.

Revised **Table 1**.

| Variables | Total  N = 2478 | Serious adverse events due to PZA | | *P* value |
| --- | --- | --- | --- | --- |
|  |  | (‒), N = 2251 | (+), N = 227 |  |
| Age (year) | 49.6±18.4 | 49.0±18.3 | 55.5±18.5 | <0.001 |
| Sex, male (%) | 1319 (53.2) | 1048 (46.6) | 111 (48.9) | 0.500 |
| Tuberculosis |  |  |  | 0.291 |
| Pulmonary | 2102 (84.8) | 1904 (84.6) | 198 (87.2) |  |
| Extrapulmonary | 376 (15.2) | 347 (15.4) | 29 (12.8) |  |
| Initial diagnosis |  |  |  | 0.524 |
| Sputum AFB | 791 (33.7) | 722 (33.9) | 69 (31.8) |  |
| TB-PCR | 1553 (66.3) | 1405 (66.1) | 148 (68.2) |  |
| Comorbidities |  |  |  |  |
| DM | 244 (9.8) | 214 (9.5) | 30 (13.2) | 0.074 |
| Malignancy | 133 (5.4) | 117 (5.2) | 16 (7.0) | 0.238 |
| Renal insufficiency | 26 (1.0) | 20 (1.0) | 4 (1.8) | 0.291 |
| Smoking^a^ |  |  |  | 0.718 |
| Never | 1069 (56.8) | 871 (56.6) | 198 (57.7) |  |
| Ex- or current | 814 (43.2) | 669 (43.4) | 145 (42.3) |  |
| Alcohol use^b^ |  |  |  | 0.553 |
| Never/social | 618 (89.7) | 473 (89.2) | 145 (91.2) |  |
| Heavy | 71 (10.3) | 57 (10.8) | 14 (8.8) |  |
| Long-term steroid use | 4 (0.2) | 4 (0.2) | 0 (0.0) | 1.000 |
| Risk factors for hepatotoxicity |  |  |  |  |
| HBs Ag (+) | 57 (4.2) | 51 (4.2) | 6 (4.0) | 0.912 |
| Anti HCV (+) | 12 (0.9) | 12 (1.0) | 0 (0.0) | 0.381 |
| Initial laboratory tests |  |  |  |  |
| AST | 22.0±11.8 | 21.8±11.1 | 24.2±16.8 | 0.090 |
| ALT | 19.4±14.6 | 19.4±14.8 | 19.6±13.5 | 0.881 |
| Treatment duration (mo.) | 8.0±3.1 | 7.9±3.1 | 9.2±3.4 | <0.001 |

AFB, acid-fast blue; TB, tuberculosis; PCR, polymerase chain reaction; DM, diabetes mellitus;

^a^Data were not recorded for 595 (24.0%) patients

^b^Data were not recorded for 1789 (72.2%) patients

Data are reported as the mean ± standard deviation and numbers (%).

(Page 8–9, Table 1 in the revised manuscript)

6. In case of EPTB, SARs analysis may be different according to the TB infection site with the involvement of vital organs other than lung. Author should specify if any association were found in present study or not?

🡪 Thank you for your insightful comment. The following table shows the relationship between the TB infection site and the occurrence of SAEs due to PZA. Among the 376 patients with extrapulmonary tuberculosis, 29 (7.7%) experienced SAEs due to PZA. Although there were no significant differences in the occurrence of SAEs between the infection sites (*P* = 0.051), patients with gastrointestinal TB were more likely to experience SAEs according to our analysis.

|  | SAE- | SAE+ |
| --- | --- | --- |
| Total | 347 (92.3) | 29 (7.7) |
| Lymph node | 151 (95.6) | 7 (4.4) |
| Pleura | 10 (100.0) | 0 (0.0) |
| Bone | 49 (90.7) | 5 (9.3) |
| Central nervous system | 8 (80.0) | 2 (20.0) |
| Gastrointestinal | 86 (89.6) | 10 (10.4) |
| Miliary | 12 (92.3) | 1 (7.7) |
| Genitourinary | 31 (88.6) | 4 (11.4) |

A total of 106 patients were diagnosed as TB pleurisy in the current study. Of these 106 patients, 96 patients were accompanied by pulmonary TB and TB pleurisy, and the remaining 10 cases had only TB pleurisy. Because we categorized the case with TB pleurisy without pulmonary TB as extrapulmonary TB, the number of pleural TB was only 10 cases in this Table.

7. Author should also provide data of drug resistance patterns other then MDR-TB (already excluded for the study), which may show some other aspects to treat the patients with older regimen with PZA or newer regimen without PZA.

🡪 Thank you for your comment. According to the 2014 WHO guideline, PZA was recommended for routine use in MDR-TB. In contrast, EMB was not recommended according to the guideline. Thus, we excluded the MDR-TB cases to investigate the SAEs due to first-line anti-TB drugs. After excluding 97 patients with MDR-TB, 40 and 3 patients with INH- and RIF-resistant (not MDR-TB), respectively, were identified. All 43 patients were administered PZA-containing first-line drug regimens at first. There was no significant difference in SAE occurrence between drug-sensitive and -resistant TB as shown in the following table.

|  | Total | SAE- | SAE+ | *P* value |
| --- | --- | --- | --- | --- |
|  |  |  |  | 0.678 |
| Drug-sensitive TB | 2435 (98.3) | 2036 (98.3%) | 399 (98.0) |  |
| INH- or RIF-resistant TB | 43 (1.7) | 35 (1.7%) | 8 (2.0) |  |
